# 2-[(4-Chloro­phen­yl)imino­meth­yl]hydro­quinone

**DOI:** 10.1107/S1600536809015396

**Published:** 2009-04-30

**Authors:** Serap Yazıcı, Ferda Erşahin, Erbil Ağar, İsmet Şenel, Orhan Büyükgüngör

**Affiliations:** a Department of Physics, Faculty of Arts and Sciences, Ondokuz Mayís University, TR-55139 Kurupelit–Samsun, Turkey; b Department of Chemistry, Faculty of Arts and Sciences, Ondokuz Mayís University, TR-55139 Kurupelit–Samsun, Turkey

## Abstract

The title compound, C_13_H_10_ClNO_2_, exists in the phenol–imine form in the crystal, and the aromatic rings are oriented at a dihedral angle of 2.82 (9)°. An intra­molecular O—H⋯N hydrogen bond results in the formation of a planar six-membered ring. In the crystal structure, inter­molecular O—H⋯O hydrogen bonds link the mol­ecules into chains.

## Related literature

For general background to *o*-hydr­oxy Schiff bases, see: Calligaris *et al.* (1972 ▶); Hadjoudis *et al.* (1987 ▶); Hökelek *et al.* (2004 ▶); Maslen & Waters (1975 ▶); Moustakali-Mavridis *et al.* (1980 ▶); Xu *et al.* (1994 ▶). For related structures, see: Filarowski *et al.* (2003 ▶); Karadayı *et al.* (2003 ▶); Yıldız *et al.* (1998 ▶).
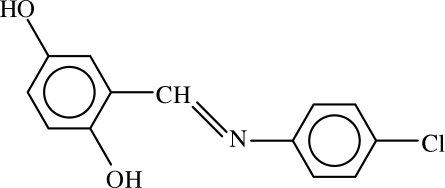


## Experimental

### 

#### Crystal data

C_13_H_10_ClNO_2_
*M* *_r_* = 247.67
Monoclinic, 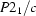

*a* = 20.3347 (14) Å
*b* = 4.5848 (2) Å
*c* = 12.0383 (9) Å
β = 98.231 (6)°
*V* = 1110.78 (12) Å^3^
*Z* = 4
Mo *K*α radiation
μ = 0.33 mm^−1^
*T* = 296 K
0.80 × 0.40 × 0.06 mm

#### Data collection

Stoe IPDS-II diffractometer
Absorption correction: integration (*X-RED32*; Stoe & Cie, 2002 ▶) *T*_min_ = 0.819, *T*_max_ = 0.978
15373 measured reflections
2185 independent reflections
1575 reflections with *I* > 2σ(*I*)
*R*_int_ = 0.069

#### Refinement

*R*[*F* ^2^ > 2σ(*F* ^2^)] = 0.034
*wR*(*F* ^2^) = 0.091
*S* = 0.95
2185 reflections
154 parameters
H-atom parameters constrained
Δρ_max_ = 0.13 e Å^−3^
Δρ_min_ = −0.25 e Å^−3^

### 

Data collection: *X-AREA* (Stoe & Cie, 2002 ▶); cell refinement: *X-RED32* (Stoe & Cie, 2002 ▶); data reduction: *X-RED32*; program(s) used to solve structure: *SHELXS97* (Sheldrick, 2008 ▶); program(s) used to refine structure: *SHELXL97* (Sheldrick, 2008 ▶); molecular graphics: *ORTEP-3 for Windows* (Farrugia, 1997 ▶); software used to prepare material for publication: *WinGX* (Farrugia, 1999 ▶).

## Supplementary Material

Crystal structure: contains datablocks I, global. DOI: 10.1107/S1600536809015396/hk2670sup1.cif

Structure factors: contains datablocks I. DOI: 10.1107/S1600536809015396/hk2670Isup2.hkl

Additional supplementary materials:  crystallographic information; 3D view; checkCIF report

## Figures and Tables

**Table 1:** Hydrogen-bond geometry (Å, °)

*D*—H⋯*A*	*D*—H	H⋯*A*	*D*⋯*A*	*D*—H⋯*A*
O1—H1⋯N1	0.82	1.90	2.6270 (18)	147
O2—H2⋯O2^i^	0.82	2.04	2.7631 (13)	147

Symmetry code: (i) 
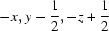
.

**Fig. 1.**
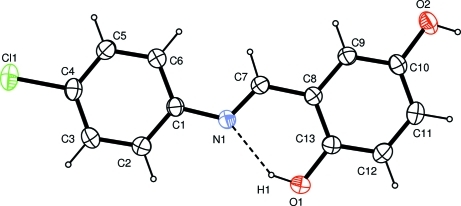
The molecular structure of the title molecule, with the atom-numbering scheme. Displacement ellipsoids are drawn at the 30% probability level. Hydrogen bonds are shown as dashed lines.

**Fig. 2.**
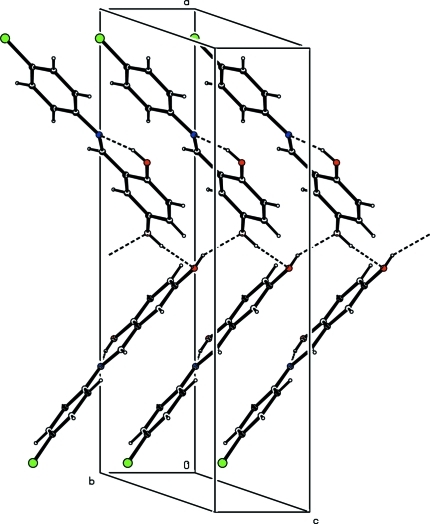
A partial packing diagram of the title compound. Hydrogen bonds are shown as dashed lines.

## supplementary crystallographic information

### Comment

*o*-Hydroxy Schiff bases derived from the reactions of *o*-hydroxyaldehydes with aniline have been examined extensively (Calligaris *et al.*, 1972; Maslen & Waters, 1975). In general, *o*-hydroxy Schiff bases exhibit two possible tautomeric forms, namely, phenol-imine and keto-amine. Naphthaldimine and salicylaldimine can also exist in the phenol-imine and keto-amine forms, respectively depending on the stereochemistry of the molecule and the type of nitrogen substituents in naphthaldimine and salicylaldimine Schiff bases (Hökelek *et al.*, 2004). Schiff base compounds display interesting photochromic and thermochromic features and can be classified in terms of these ( Moustakali-Mavridis *et al.*, 1980; Hadjoudis *et al.*, 1987). Photo- and thermochromism arise *via* H atom transfer from the hydroxy O atom to the N atom (Hadjoudis *et al.*, 1987; Xu *et al.*, 1994).

In the title compound (Fig. 1), the phenol-imine form is favored over the keto-amine form, as indicated by C13-O1 [1.356 (2) Å] and C7-N1 [1.280 (2) Å] bonds, which are in accordance with the corresponding values in a similar compound [C-O = 1.352 (3) and C-N = 1.280 (4) Å; Karadayı *et al.*, 2003]. As a common feature of *o*-hydroxysalicylidene systems, the title compound displays a strong hydrogen bond between atoms N1 and O1 (Filarowski *et al.*, 2003; Yıldız *et al.*, 1998).

It is known that Schiff bases may exhibit thermochromism or photochromism, depending on the planarity or non-planarity of the molecule, respectively. Therefore, one can expect thermochromic properties in the title compound caused by planarity of the molecule; the dihedral angle between rings A (C1-C6) and B (C8-C13) is 2.82 (9)°. Intramolecular O-H···N hydrogen bond (Table 1) results in the formation of a planar six-membered ring C (O1/N1/C7/C8/C13/H1), which is oriented with respect to rings A and B at dihedral angles of A/C = 2.97 (8) and B/C = 1.35 (8) °. So, they are nearly coplanar.

In the crystal structure, intermolecular O-H···O hydrogen bonds (Table 1) link the molecules into chains (Fig. 2), in which they may be effective in the stabilization of the structure.

### Experimental

The title compound was prepared by refluxing a mixture of a solution containing 2,5-dihydroxybenzaldehyde (0.034 g 0.246 mmol) in ethanol (20 ml) and a solution containing 4-chloroaniline (0.031 g 0.246 mmol) in ethanol (20 ml). The reaction mixture was stirred for 1 h under reflux. Crystals suitable for X-ray analysis were obtained from ethylalcohol by slow evaporation (yield; 69%; m.p. 439-441 K).

### Refinement

H atoms were positioned geometrically, with O-H = 0.82 Å (for OH) and C-H = 0.93 Å for aromatic H, respectively, and constrained to ride on their parent atoms, with U_iso_(H) = xU_eq_(C,O), where x = 1.5 for OH H and x = 1.2 for aromatic H atoms.

### Crystal data

**Table:**

C_13_H_10_ClNO_2_	*F*(000) = 512
*M**_r_* = 247.67	*D*_x_ = 1.481 Mg m^−^^3^
Monoclinic, *P*2_1_/*c*	Mo *K*α radiation, λ = 0.71073 Å
Hall symbol: -P 2ybc	Cell parameters from 13223 reflections
*a* = 20.3347 (14) Å	θ = 1.7–27.2°
*b* = 4.5848 (2) Å	µ = 0.33 mm^−^^1^
*c* = 12.0383 (9) Å	*T* = 296 K
β = 98.231 (6)°	Plate, brown
*V* = 1110.78 (12) Å^3^	0.80 × 0.40 × 0.06 mm
*Z* = 4	

### Data collection

**Table:**

Stoe IPDS-II diffractometer	2185 independent reflections
Radiation source: fine-focus sealed tube	1575 reflections with *I* > 2σ(*I*)
graphite	*R*_int_ = 0.069
Detector resolution: 6.67 pixels mm^-1^	θ_max_ = 26.0°, θ_min_ = 2.0°
ω scans	*h* = −25→25
Absorption correction: integration (*X-RED32*; Stoe & Cie, 2002)	*k* = −5→5
*T*_min_ = 0.819, *T*_max_ = 0.978	*l* = −14→14
15373 measured reflections	

### Refinement

**Table:**

Refinement on *F*^2^	Primary atom site location: structure-invariant direct methods
Least-squares matrix: full	Secondary atom site location: difference Fourier map
*R*[*F*^2^ > 2σ(*F*^2^)] = 0.034	Hydrogen site location: inferred from neighbouring sites
*wR*(*F*^2^) = 0.091	H-atom parameters constrained
*S* = 0.95	*w* = 1/[σ^2^(*F*_o_^2^) + (0.0571*P*)^2^] where *P* = (*F*_o_^2^ + 2*F*_c_^2^)/3
2185 reflections	(Δ/σ)_max_ < 0.001
154 parameters	Δρ_max_ = 0.13 e Å^−^^3^
0 restraints	Δρ_min_ = −0.24 e Å^−^^3^

### Special details

**Table:**

Experimental. 370 frames, detector distance = 120 mm
Geometry. All e.s.d.'s (except the e.s.d. in the dihedral angle between two l.s. planes) are estimated using the full covariance matrix. The cell e.s.d.'s are taken into account individually in the estimation of e.s.d.'s in distances, angles and torsion angles; correlations between e.s.d.'s in cell parameters are only used when they are defined by crystal symmetry. An approximate (isotropic) treatment of cell e.s.d.'s is used for estimating e.s.d.'s involving l.s. planes.
Refinement. Refinement of *F*^2^ against ALL reflections. The weighted *R*-factor *wR* and goodness of fit *S* are based on *F*^2^, conventional *R*-factors *R* are based on *F*, with *F* set to zero for negative *F*^2^. The threshold expression of *F*^2^ > σ(*F*^2^) is used only for calculating *R*-factors(gt) *etc*. and is not relevant to the choice of reflections for refinement. *R*-factors based on *F*^2^ are statistically about twice as large as those based on *F*, and *R*- factors based on ALL data will be even larger.

### Fractional atomic coordinates and isotropic or equivalent isotropic displacement parameters (Å^2^)

**Table:**

	*x*	*y*	*z*	*U*_iso_*/*U*_eq_	
Cl1	0.46483 (2)	1.40871 (10)	0.33464 (4)	0.06076 (18)	
O1	0.21937 (7)	0.2322 (3)	0.60021 (10)	0.0600 (4)	
H1	0.2441	0.3500	0.5759	0.090*	
O2	0.03814 (6)	−0.1943 (3)	0.25297 (11)	0.0554 (3)	
H2	0.0127	−0.3115	0.2757	0.083*	
N1	0.26643 (6)	0.5541 (3)	0.44912 (11)	0.0404 (3)	
C1	0.31206 (7)	0.7617 (3)	0.41651 (14)	0.0390 (3)	
C2	0.35972 (8)	0.8701 (4)	0.50029 (14)	0.0469 (4)	
H2A	0.3599	0.8079	0.5739	0.056*	
C3	0.40681 (8)	1.0686 (4)	0.47659 (15)	0.0491 (4)	
H3	0.4388	1.1380	0.5334	0.059*	
C4	0.40585 (8)	1.1623 (3)	0.36807 (15)	0.0445 (4)	
C5	0.35838 (9)	1.0629 (4)	0.28379 (15)	0.0499 (4)	
H5	0.3578	1.1304	0.2108	0.060*	
C6	0.31162 (8)	0.8628 (4)	0.30758 (14)	0.0480 (4)	
H6	0.2796	0.7954	0.2504	0.058*	
C7	0.22399 (8)	0.4302 (3)	0.37519 (13)	0.0409 (4)	
H7	0.2238	0.4777	0.3000	0.049*	
C8	0.17625 (7)	0.2186 (3)	0.40433 (13)	0.0384 (3)	
C9	0.13019 (8)	0.1001 (3)	0.31857 (14)	0.0421 (4)	
H9	0.1312	0.1561	0.2446	0.051*	
C10	0.08364 (8)	−0.0975 (3)	0.34244 (14)	0.0427 (4)	
C11	0.08231 (9)	−0.1840 (4)	0.45228 (16)	0.0510 (4)	
H11	0.0504	−0.3164	0.4686	0.061*	
C12	0.12807 (9)	−0.0745 (4)	0.53729 (15)	0.0530 (4)	
H12	0.1274	−0.1360	0.6107	0.064*	
C13	0.17529 (8)	0.1273 (4)	0.51433 (13)	0.0436 (4)	

### Atomic displacement parameters (Å^2^)

**Table:**

	*U*^11^	*U*^22^	*U*^33^	*U*^12^	*U*^13^	*U*^23^
Cl1	0.0545 (3)	0.0499 (3)	0.0821 (4)	−0.0131 (2)	0.0244 (2)	−0.0049 (2)
O1	0.0675 (8)	0.0733 (8)	0.0374 (7)	−0.0226 (7)	0.0010 (6)	0.0007 (6)
O2	0.0450 (7)	0.0519 (7)	0.0651 (8)	−0.0090 (5)	−0.0062 (6)	−0.0033 (6)
N1	0.0395 (7)	0.0397 (7)	0.0417 (7)	−0.0030 (6)	0.0047 (6)	−0.0009 (6)
C1	0.0380 (8)	0.0355 (7)	0.0436 (9)	−0.0002 (6)	0.0065 (7)	−0.0012 (7)
C2	0.0499 (9)	0.0477 (9)	0.0426 (9)	−0.0046 (7)	0.0049 (7)	−0.0003 (7)
C3	0.0448 (9)	0.0481 (9)	0.0527 (11)	−0.0080 (8)	0.0012 (7)	−0.0062 (8)
C4	0.0402 (9)	0.0367 (8)	0.0590 (11)	−0.0017 (6)	0.0148 (8)	−0.0036 (7)
C5	0.0546 (10)	0.0507 (9)	0.0457 (10)	−0.0048 (8)	0.0115 (8)	0.0037 (8)
C6	0.0469 (9)	0.0514 (10)	0.0446 (10)	−0.0094 (7)	0.0025 (7)	0.0003 (8)
C7	0.0436 (8)	0.0408 (8)	0.0383 (9)	−0.0014 (7)	0.0062 (7)	0.0019 (7)
C8	0.0380 (8)	0.0371 (8)	0.0400 (9)	0.0013 (6)	0.0050 (7)	−0.0007 (6)
C9	0.0445 (9)	0.0414 (8)	0.0398 (9)	0.0004 (7)	0.0036 (7)	0.0026 (7)
C10	0.0360 (8)	0.0390 (8)	0.0514 (10)	0.0006 (7)	0.0008 (7)	−0.0037 (7)
C11	0.0454 (10)	0.0472 (9)	0.0622 (12)	−0.0074 (7)	0.0141 (8)	0.0023 (8)
C12	0.0591 (11)	0.0559 (10)	0.0458 (10)	−0.0076 (9)	0.0138 (8)	0.0056 (8)
C13	0.0444 (9)	0.0474 (9)	0.0391 (9)	−0.0027 (7)	0.0060 (7)	−0.0019 (7)

### Geometric parameters (Å, °)

**Table:**

O1—H1	0.8200	C7—N1	1.280 (2)
O2—H2	0.8200	C7—C8	1.451 (2)
C1—C2	1.387 (2)	C7—H7	0.9300
C1—C6	1.390 (2)	C8—C13	1.392 (2)
C1—N1	1.4229 (19)	C8—C9	1.401 (2)
C2—C3	1.380 (2)	C9—C10	1.370 (2)
C2—H2A	0.9300	C9—H9	0.9300
C3—C4	1.373 (3)	C10—C11	1.384 (2)
C3—H3	0.9300	C10—O2	1.3883 (19)
C4—C5	1.374 (2)	C11—C12	1.376 (3)
C4—Cl1	1.7365 (16)	C11—H11	0.9300
C5—C6	1.381 (2)	C12—C13	1.389 (2)
C5—H5	0.9300	C12—H12	0.9300
C6—H6	0.9300	C13—O1	1.3557 (19)
			
C13—O1—H1	109.5	N1—C7—C8	122.44 (14)
C10—O2—H2	109.5	N1—C7—H7	118.8
C7—N1—C1	120.41 (14)	C8—C7—H7	118.8
C2—C1—C6	118.42 (15)	C13—C8—C9	119.06 (15)
C2—C1—N1	117.03 (14)	C13—C8—C7	122.16 (14)
C6—C1—N1	124.55 (14)	C9—C8—C7	118.78 (14)
C3—C2—C1	121.29 (16)	C10—C9—C8	120.75 (15)
C3—C2—H2A	119.4	C10—C9—H9	119.6
C1—C2—H2A	119.4	C8—C9—H9	119.6
C4—C3—C2	119.16 (16)	C9—C10—C11	119.88 (15)
C4—C3—H3	120.4	C9—C10—O2	116.90 (15)
C2—C3—H3	120.4	C11—C10—O2	123.20 (15)
C3—C4—C5	120.78 (15)	C12—C11—C10	120.16 (16)
C3—C4—Cl1	120.53 (13)	C12—C11—H11	119.9
C5—C4—Cl1	118.69 (14)	C10—C11—H11	119.9
C4—C5—C6	119.93 (16)	C11—C12—C13	120.54 (16)
C4—C5—H5	120.0	C11—C12—H12	119.7
C6—C5—H5	120.0	C13—C12—H12	119.7
C5—C6—C1	120.39 (15)	O1—C13—C12	118.99 (15)
C5—C6—H6	119.8	O1—C13—C8	121.41 (14)
C1—C6—H6	119.8	C12—C13—C8	119.59 (15)
			
C8—C7—N1—C1	−179.65 (13)	N1—C7—C8—C9	178.06 (15)
C2—C1—N1—C7	−175.30 (14)	C13—C8—C9—C10	1.7 (2)
C6—C1—N1—C7	5.2 (2)	C7—C8—C9—C10	−179.12 (14)
C6—C1—C2—C3	−1.5 (2)	C8—C9—C10—C11	−0.7 (2)
N1—C1—C2—C3	178.92 (14)	C8—C9—C10—O2	177.31 (14)
C1—C2—C3—C4	0.7 (3)	C9—C10—C11—C12	−0.7 (3)
C2—C3—C4—C5	0.6 (3)	O2—C10—C11—C12	−178.57 (15)
C2—C3—C4—Cl1	−179.27 (12)	C10—C11—C12—C13	1.1 (3)
C3—C4—C5—C6	−1.1 (3)	C11—C12—C13—O1	179.57 (16)
Cl1—C4—C5—C6	178.81 (13)	C11—C12—C13—C8	−0.1 (3)
C4—C5—C6—C1	0.2 (3)	C9—C8—C13—O1	179.08 (14)
C2—C1—C6—C5	1.1 (2)	C7—C8—C13—O1	−0.1 (2)
N1—C1—C6—C5	−179.43 (15)	C9—C8—C13—C12	−1.3 (2)
N1—C7—C8—C13	−2.7 (2)	C7—C8—C13—C12	179.54 (15)

### Hydrogen-bond geometry (Å, °)

**Table:**

*D*—H···*A*	*D*—H	H···*A*	*D*···*A*	*D*—H···*A*
O1—H1···N1	0.82	1.90	2.6270 (18)	147
O2—H2···O2^i^	0.82	2.04	2.7631 (13)	147

Symmetry codes: (i) −*x*, *y*−1/2, −*z*+1/2.
